# Transcriptome Sequencing Unveils a Novel Mechanism Underlying Breed Distinctions Between Thin- and Fat-Tailed Sheep

**DOI:** 10.3390/genes17020162

**Published:** 2026-01-30

**Authors:** Lei Gao, Yunyun Zhang, Yiyuan Zhang, Weifeng Peng, Zhenliang Zhang, Yucheng Liu, Jingjing Wang, Pengcheng Wan, Zongsheng Zhao

**Affiliations:** 1 College of Animal Science and Technology, Shihezi University, Shihezi 832000, China; w.n007@163.com; 2 State Key Laboratory of Sheep Genetic Improvement and Healthy Production, Xinjiang Academy of Agricultural and Reclamation Science, Shihezi 832000, China; 13838997301@163.com (Y.Z.); yyzhang1876@163.com (Y.Z.); zhangzhenliang27@163.com (Z.Z.); 15299438310@163.com (Y.L.); 13999533801@163.com (J.W.); 3 College of Life Science and Agronomy, Zhoukou Normal University, Zhoukou 466000, China; pengwf226@163.com

**Keywords:** sheep, transcriptome sequencing, LncRNA, miRNA, fat deposition

## Abstract

Background: Sheep (*Ovis aries*) tail fat serves as a crucial energy reserve for adapting to harsh environments. However, excessive deposition can reduce farming efficiency and product quality. Elucidating the regulatory mechanisms of tail fat deposition is of great significance for genetic improvement in sheep. Methods: In this study, transcriptome sequencing was conducted on tail fat tissues from fat-tailed Kazakh sheep (KAZ), thin-tailed Suffolk sheep (SFK), and their F2 hybrid sheep (CSH) (3 individuals per group). Subsequently, qRT-PCR validation, Enrichr, and KEGG database analyses were performed to investigate the molecular pathways involved in tail fat deposition. Results: High-quality clean reads were obtained from sequencing, with a genome alignment rate ranging from 76.15% to 79.43% and good data reproducibility. Differential expression analysis revealed multiple differentially expressed genes (DEGs) between KAZ and CSH groups, KAZ and SFK groups, as well as SFK and CSH groups. Five core candidate genes (*BDH1*, *EPHX1*, *BCAT2*, *FASN*, *ACACA*) were identified, all enriched in the fatty acid synthesis pathway and highly expressed in fat-tailed sheep, which was confirmed by qRT-PCR. Additionally, 189 lncRNAs were identified to collectively regulate target genes (e.g., *FABP* family, *AGPAT2*), along with three common differentially expressed miRNAs (novel_120, novel_171, novel_440) targeting genes enriched in lipid transport and lipid droplet formation pathways. Conclusions: This study confirms that the lncRNA-mRNA-miRNA regulatory axis is a key pathway in tail fat formation, providing important theoretical support and molecular targets for genetic improvement of ovine tail fat deposition traits.

## 1. Introduction

Sheep (*Ovis aries*) are among the earliest domesticated livestock and play a crucial role in the global agricultural economy by providing meat, wool, milk, and leather [1,2]. Among the various economic traits of sheep, fat deposition characteristics are not only key factors determining meat production performance and meat quality but also core physiological processes for adapting to different environments and maintaining energy balance [3,4]. Sheep fat can be categorized into different types including subcutaneous fat, visceral fat, intramuscular fat, and tail fat, depending on its anatomical position and physiological role [5,6]. The formation and regulation mechanism of tail fat has always been a hot topic in animal genetics and physiology research. Many sheep breeds around the world, especially those cultivated in dry and semi-arid regions of Central Asia, the Middle East, and Africa, have the ability to deposit large amounts of fat in their tails or rumps, forming a unique fat-tail or fat-rump phenotype [7]. This unique fat depot is considered to be an energy storage strategy evolved by sheep to adapt to the harsh environment of food scarcity and drastic seasonal changes during long-term natural and artificial selection processes [8,9]. During periods of drought or migration when energy demands are high, tail fat can be broken down and utilized to provide essential energy and hydration to the body, greatly enhancing the survival ability of sheep [10]. However, as modern animal husbandry moves towards intensification and scale, and consumer preference for low-fat, high-quality meat increases, excessive tail fat deposition has gradually become an economic burden [11]. The large tail fat not only reduces slaughter rate and net meat yield, but also increases feed costs, and has a commercial value far lower than muscle tissue [10]. Therefore, reducing tail fat deposition through genetic improvement while maintaining the excellent adaptability of sheep is one of the important goals in sheep breeding [12]. To achieve this goal, it is necessary to have a deep understanding of the molecular genetic mechanisms regulating tail fat development and fat deposition.

The transcriptome refers to the total sum of all RNA molecules transcribed in a specific cell or tissue under a specific state, mainly including messenger RNA (mRNA), ribosomal RNA (rRNA), transfer RNA (tRNA), and various non-coding RNA (ncRNA) [13]. Transcriptome serves as a crucial bridge connecting the genome (genetic information) and the proteome (functional executors), with changes in its expression pattern directly reflecting alterations in gene activity and determining the physiological state and function of cells [14]. Therefore, transcriptome analysis has become a core approach to uncovering the molecular mechanisms underlying complex biological processes such as development, disease, and stress responses [15,16]. Thanks to the rapid development of transcriptomics technology, scholars both domestically and internationally have conducted a large amount of research on the molecular mechanisms of tail fat deposition in sheep [17,18], achieving significant progress. These studies mainly employ a comparative transcriptomics strategy, comparing gene expression profiles of different tail types (fat-tailed vs. thin-tailed), different developmental stages, or different fat depots to screen for candidate genes and signaling pathways related to tail fat deposition. Early studies focused on using RNA-Seq technology to identify differentially expressed genes (DEGs) in the tail fat tissues of fat-tailed and thin-tailed sheep [8,9]. A study comparing the transcriptomes of Kazakh sheep (fat-tailed) and Tibetan sheep (short-tailed) identified 646 DEGs. Pathway analysis revealed enrichment of these genes in pathways related to fat digestion and absorption, amino acid metabolism, and cell adhesion [19]. Another study compared the Lori-Bakhtiari (fat-tailed) and Zel (thin-tailed) breeds in Iran, identifying a total of 264 DEGs. Functional enrichment analysis showed that, in addition to lipid metabolism pathways, interleukin response, MAPK signaling pathway, Wnt signaling pathway, and extracellular matrix (ECM)-receptor interaction may also be involved in regulating tail fat deposition [20]. As research progresses, scholars are not only focusing on differences between different breeds, but also exploring the transcriptome characteristics of different fat depots within the same breed. A study comparing the subcutaneous, visceral, and tail fat tissues of Tan sheep found significant differences in gene expression among the different depots. The tail fat tissue specifically expressed multiple *HOX* family genes (such as *HOXC11*, *HOXC12*, *HOXC13*), suggesting that these developmental regulatory genes may play important roles in the formation of tail fat specificity [6]. The research on Guangling Big-Tailed Sheep and Hanzhong Small-Tailed Sheep also revealed significant differences in the number and functions of DEGs in different fat depots (perirenal, subcutaneous, tail), the PPAR signaling pathway and the ECM-receptor interaction pathway were significantly enriched in multiple comparison groups, indicating that they are core pathways regulating fat deposition [5].

Besides mRNA responsible for protein synthesis, the transcriptome comprises a substantial quantity of non-coding RNAs (ncRNAs) that possess crucial regulatory functions, including long non-coding RNAs (lncRNAs) and microRNAs (miRNAs) [21]. These ncRNAs regulate the expression of target genes at multiple levels, including transcription, post-transcription, and epigenetics, and are involved in almost all life processes [22,23,24]. In recent years, there has been a gradual increase in research on ncRNA in sheep tail fat. A study identified 728 differentially expressed lncRNAs in the tail fat tissue of Bashbay sheep (fat-tailed) and their hybrid offspring (thin-tailed) through whole transcriptome sequencing, and found that two lncRNAs may affect fat deposition by regulating their cis-target genes *FASN* and *THRSP* [25]. Another study identified lncRNAs in sheep tail fat for the first time and constructed a lncRNA-mRNA co-expression network, revealing modules related to lipid metabolism, insulin, and calcium signaling pathways [20]. In the study of miRNAs, scientists compared the tail fat miRNA expression profiles of Hu sheep (short fat tail) and Tibetan sheep (short thin tail), identifying 155 differentially expressed miRNAs. Through experimental validation, they confirmed the regulatory relationship between miR-379-5p and its target gene *HOXC9*, uncovering the role of miRNA in tail fat deposition [26].

Although significant progress has been made in current research uncovering the molecular mechanisms of sheep tail fat deposition, there are still some knowledge gaps and challenges that need to be addressed by future studies. In this study, we performed transcriptome sequencing of caudal adipose tissue from Suffolk (SFK) (a thin-tailed breed), Kazakh (KAZ) (a fat-tailed breed) and the F2 generation of Suffolk × Kazakh (CSH). Our goal was to identify differentially expressed coding and non-coding RNAs and to elucidate regulatory networks and metabolic pathways involved in tail fat deposition. By characterizing key miRNAs, lncRNAs, and their target genes, this work aims to advance our understanding of the molecular mechanisms governing adipogenesis in the sheep tail, providing insights with potential applications in sheep breeding and genetic improvement.

## 2. Materials and Methods

### 2.1. Sample Preparation

**Figure 1:**
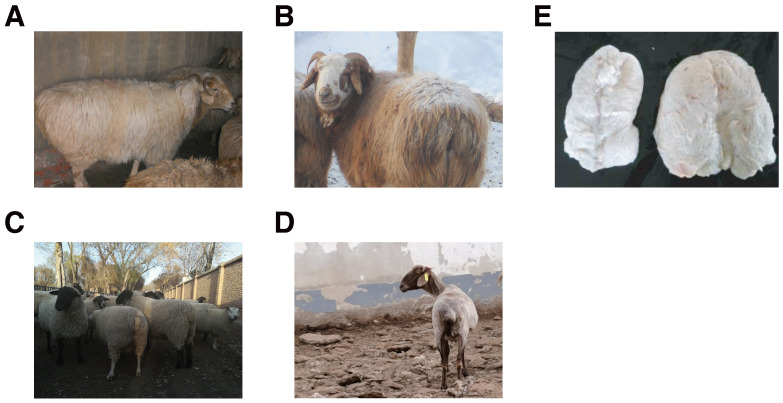
Different phenotypes of the sheep (*Ovis aries*) and its fat deposition in tail. (**A**,**B**) The Kazakh sheep, fat-tailed sheep, is characterized by an increased propensity for fat deposition in the tails. (**C**) Suffolk sheep, thin-tailed sheep, is endowed with a diminutive tail size. (**D**) F2 generation of Kazakh and Suffolk sheep, exhibit a thin tail characteristic. However, they demonstrate a higher deposition rate in comparison to Suffolk sheep. (**E**) The adipose tissues of the F2 and Kazakh sheep breeds were analyzed. The left side corresponds to the F2, while the right side is representative of the Kazakh sheep.

In this study, three healthy adult KAZ ewes (fat-tailed), three CSH, and three adult SFK rams (lean-tailed) were selected as experimental subjects (Figure 1). All animals were approximately 18 months of age and raised under uniform feeding and management conditions at the Ashan Tunke Meat Sheep Breeding Co., Ltd. in Beitun, Xinjiang, China (affiliated with the Xinjiang Academy of Agricultural and Reclamation Sciences). Sample collection was performed in December, the period of peak tail fat deposition in sheep. Before sampling, the tail region was sterilized using 75% ethanol followed by 5% iodine tincture. Local anesthesia was administered via multi-point injection at the surgical site. After a 3–5 min observation to ensure a stable physiological condition, a small surgical incision was made to collect subcutaneous caudal adipose tissue. Each sample consisted of approximately 2–3 g of tail fat. All instruments used were sterilized and RNase-free to ensure sample integrity for transcriptomic analysis. The procedure was completed within 10 min per animal to minimize stress, and all wounds were sutured and disinfected immediately post-operation. Collected fat tissues were rapidly frozen in liquid nitrogen and stored at –80 °C for RNA extraction. The entire procedure was performed in accordance with institutional ethical standards and with the formal consent of the sheep breeding facility.

### 2.2. RNA Isolation

RNA was extracted from tail tissues, and the samples underwent stringent quality control based on the following criteria. First, agarose gel electrophoresis was used to assess RNA integrity and detect potential DNA contamination. Second, RNA concentration and purity were preliminarily quantified. Finally, the integrity of RNA was accurately measured.

### 2.3. Quantitative Real-Time PCR (qRT-PCR)

Quantitative real-time PCR was performed using cDNA from tail fat tissues of KAZ and SFK sheep, with *GAPDH* as the internal reference gene [27]. The ovine *FASN*, *BCAT2*, *BDH1*, *ACACA*, and *EPHX1* mRNA sequences were retrieved from the Ensembl Genome Browser. Specific primers were designed using Oligo 6.0 software and synthesized by Youkang Biotechnology Co., Ltd. (Shanghai, China). Primer information is provided in Table S1. Each sample was analyzed in triplicate with appropriate negative controls. The amplification protocol was: 95 °C for 30 s, followed by 45 cycles of 95 °C for 5 s, 60 °C for 20 s, and 72 °C for 20 s. Dissociation curve analysis was performed at a ramp rate of 0.1 °C/s to confirm specificity. SPSS 22.0 software (IBM Corporation, 1 New Orchard Road, Armonk, NY 10504, USA). One-way ANOVA and independent sample *t*-test was used to compare variables between treatments. Information about checking of normality distribution data in each treatment using Shapiro-Wilk’s test and homogeneity of variance between treatments (Levene’s test before one-way ANOVA and F-test in case of *t*-test).

### 2.4. Library Preparation

For library construction, strand-specific libraries were prepared using a ribosomal RNA (rRNA) depletion method. Total RNA was first treated to remove rRNA, and the remaining RNA was fragmented into short segments of 250–300 bp. These fragments served as templates for first-strand cDNA synthesis using random oligonucleotide primers. The second-strand cDNA was then synthesized using dNTPs as substrates. The double-stranded cDNA was purified, end-repaired, A-tailed, and ligated with sequencing adapters. The second strand of cDNA was degraded using the USER enzyme, and the remaining cDNA was amplified by PCR to obtain the final strand-specific library. To ensure library quality, a Qubit fluorometer was used for preliminary quantification, diluting the library to 1 ng/µL. The Agilent 2100 Bioanalyzer was then employed to assess the insert fragment size, which was expected to range from 250 to 300 bp. After confirming the expected insert size, qRT-PCR was conducted for precise quantification of the library’s effective concentration, which was required to exceed 2 nM to ensure high-quality samples.

Finally, libraries that passed quality control were pooled based on their effective concentration and sequencing data requirements for Illumina PE150 sequencing. In paired-end sequencing (PE150), each end of the cDNA insert is sequenced to a length of 150 bp, with the insert fragment serving as the sequencing unit. Paired-end sequencing not only provides sequence information from both ends of the insert fragment but also offers insights into the length between the two ends, facilitating downstream assembly and alignment.

### 2.5. RNA Sequencing

The sequencing was performed using the Sequencing by Synthesis (SBS) method. In this approach, the flow cell was loaded with four fluorescently labeled dNTPs, DNA polymerase, and adapter primers. During each cycle of extension, a fluorescently labeled dNTP was incorporated into the complementary DNA strand, releasing a fluorescence signal specific to the incorporated nucleotide. These signals were captured by the sequencer, and computational software converted the fluorescence data into sequencing peaks, providing the nucleotide sequence of the target fragments.

### 2.6. Quality Control, Mapping and Quantification

Quality control and trimming/filtering of raw sequencing reads were performed using FastQC (v0.12.1) [28] and Trimmomatic (v0.35) [29] software. Raw reads containing adapter contamination, more than 10% of unknown bases, or more than 50% of low-quality bases were removed. The clean reads were aligned to sheep reference genome (Oar_v4.0) using Bowtie2 (v2.3.4) [30] for initial read mapping, followed by TopHat (v2.1.1) [31] for splice junction-aware alignment to account for RNA splicing events. SAMtools (v1.3.1) [32] was used to convert the resulting SAM (Sequence Alignment/Map) files to sorted BAM (Binary Alignment/Map) format and generate index files (.bai) for efficient downstream analysis. Assembled transcripts from the aligned reads were annotated with the NCBI reference annotation using Cufflinks (v2.2.1) [33], producing annotated gene/transcript files in GTF (Gene Transfer Format).

### 2.7. Data Analysis

FPKM normalization [34] was applied to mapped read counts to account for variations in both gene length and library size. FPKM values from 9 sheep samples were used to perform the PCA using OmicShare tools. Genes were classified as differentially expressed genes (DEGs) if they met the thresholds of |log_2_(fold change)| ≥ 2 and adjusted *p*-value ≤ 0.05 [35] when comparing fat-tailed/fat-rumped individuals with thin-tailed counterparts. For functional annotation of DEGs, a web-based platform was employed. Specifically, the Enrichr database (https://amp.pharm.mssm.edu/Enrichr (accessed on 15 May 2025)) was used to perform functional enrichment analysis on the identified DEGs, while Kyoto Encyclopedia of Genes and Genomes (KEGG) pathway analysis was conducted via its official database (http://www.genome.jp/kegg/ (accessed on 20 May 2025)). GO terms and KEGG pathways were regarded as significantly enriched when their respective adjusted *p*-values were less than 0.05.

We used the Cufflinks (v2.1.1) to merge the transcripts obtained from each sample, filtering out transcripts with ambiguous strand orientation and those shorter than 200 nt [36]. Subsequently, we compared the merged transcripts to a known database using Cuffcompare, removing transcripts already annotated in the database. Next, we predicted the coding potential of the remaining novel transcripts to obtain Novel_lncRNAs and Novel_mRNAs. The screening thresholds for mRNA and lncRNA were adjusted *p* value < 0.05 and |log fold change (FC)| > 2 [37]. The steps were as follows [38]: Step 1. Transcript exon number filtering: Discarding low-confidence single-exon transcripts and selecting transcripts with ≥2 exons. Step 2. Transcript length filtering: Choosing transcripts longer than 200 nt. Step 3. Known annotation filtering: Using Cuffcompare to eliminate transcripts overlapping with annotated exons in the database, incorporating database-annotated lncRNAs overlapping with the exons of the assembled transcripts into subsequent analyses. Step 4. Coding potential filtering: Determining coding potential is crucial for identifying lncRNAs. For the transcripts obtained in the previous step, we integrated popular coding potential analysis tools (CPC2/Pfam/CNCI) to predict coding potential. The intersection of transcripts identified as non-coding by these tools formed the candidate Novel_lncRNA dataset, while the intersection of transcripts predicted as coding formed the Novel_mRNA dataset. Step 5. Following the naming guidelines for long non-coding RNAs provided by the HUGO Gene Nomenclature Committee (HGNC), we performed final selection and naming of the candidate Novel_lncRNAs based on their positional relationship with coding genes, resulting in the Novel_lncRNAs analyzed in this study.

Identification of known miRNAs was conducted by aligning reads obtained from sequencing with sequences of specified species in the miRBase database [39]. Unannotated Clean Reads were aligned with non-coding RNA sequences in the Rfam (13.0) database [40] to annotate rRNA, tRNA, snRNA, snoRNA, and other non-coding RNAs. Unidentified miRNAs, non-coding RNAs, and repetitive sequences in Clean Reads were annotated by matching with genomic exon and intron positions (requiring 100% positional overlap) to identify small RNAs derived from mRNA. To predict potential novel miRNAs, miREvo and miRDeep2 (v2.0.0.3) software were employed [41,42]. Information on Clean Reads matching each predicted novel miRNA was obtained, followed by secondary structure and expression analysis of the predicted novel miRNAs. The screening conditions for mRNA, and miRNA, differential expression were |log FC| > 2, FDR < 0.05 [43].

## 3. Results

### 3.1. Sequencing and Mapping

Nine cDNA sequencing libraries were constructed, with three individuals each selected from the KAZ, CSH, and SFK groups. High-throughput sequencing of these libraries yielded nine datasets of reads, all with a consistent length of 150 base pairs (bp). Following quality control to remove low-quality raw reads, a total of 47,444,281, 47,346,221, 47,380,519, 47,343,113, 46,952,275, 47,046,297, 47,444,281, 47,346,221, and 47,380,519 high-quality clean reads were generated for the nine samples (KAZ1, KAZ2, KAZ3, CSH1, CSH2, CSH3, SFK1, SFK2, and SFK3), respectively, with detailed statistics provided in Table S2. Approximately 76.15–79.43% of the clean reads were aligned to the sheep reference genome, while 55.75–63.13% mapped to known reference genes. Among all clean reads, 52.45–57.26% exhibited perfect alignment to the sheep reference genome without any mismatches, 64.31–71.42% showed unique mappings, and 7–11.85% displayed multi-location mappings; the proportion of unmapped reads ranged from 20.54% to 23.83%. Regarding known sheep reference genes, 42.5–47.45% of the clean reads achieved 100% sequence identity, 51.52–57.21% had a single mapping, and 2.61–5.92% of the clean reads showed multiple mappings (Table S2).

### 3.2. Identification of Differentially Expressed Genes

**Figure 2:**
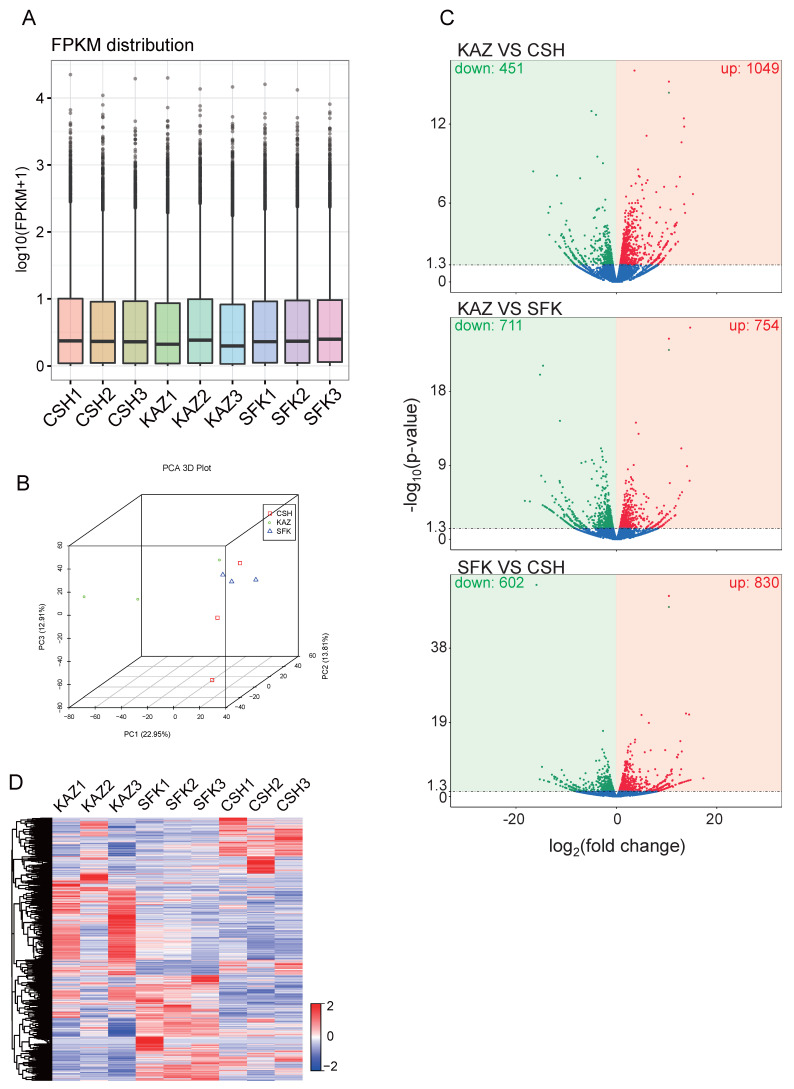
Transcriptome-wide analysis based on the differential fat deposition groups. (**A**) FPKM distribution levels are indicative of RNA expression levels in the tails’ tissues across different samples. (**B**) PCA of 9 samples. (**C**) The volcano plot is employed to illustrate the gene expression between the parents and the F2 or within those patents. The red segments of the plot denote up-regulated genes, while the green segments represent down-regulated genes. The *X*-axis denotes the fold change, while the *Y*-axis indicates the *p*-value. (**D**) The heatmap illustrates the variance in RNA levels among the sheep. The red denotes elevated levels, whereas the blue indicates reduced levels.

To investigate the regulatory mechanisms underlying tail fat deposition, RNA sequencing (RNA-seq) was employed to monitor gene expression dynamics in tail fat tissues of KAZ, SFK, and CSH. Fragments per kilobase of transcript per million mapped reads (FPKM) values were calculated, and the distribution of FPKM exhibited a relatively consistent average expression level across samples (Figure 2A). PCA was used to analyze the expression of all genes (Figure 2B). The samples from KAZ, SFK, and SFK overlapped significantly based on the PCA results. Subsequently, gene expression levels were compared among the three groups to identify significantly up-regulated and down-regulated genes (DEGs) (Figure 2C). Specifically, 1049 up-regulated and 451 down-regulated genes were detected in KAZ compared to CSH. In the KAZ vs. SFK comparison, 754 genes were significantly up-regulated and 711 were down-regulated. When comparing SFK with CSH, 830 up-regulated and 602 down-regulated genes were identified. As previously described, potential regulatory genes associated with tail fat deposition were screened by analyzing DEGs between fat-tailed (KAZ, SFK) and thin-tailed (CSH) sheep (Figure 2D). Following filtration based on the criteria of |log_2_(fold change)| ≥ 2 and adjusted *p*-value ≤ 0.05, a total of 194 genes were identified in the KAZ vs. CSH comparison group (Table S3), 214 genes in the KAZ vs. SFK comparison group (Table S4), and 249 genes in the SFK vs. CSH comparison group for differentially expressed genes (DEGs) (Table S5).

**Figure 3:**
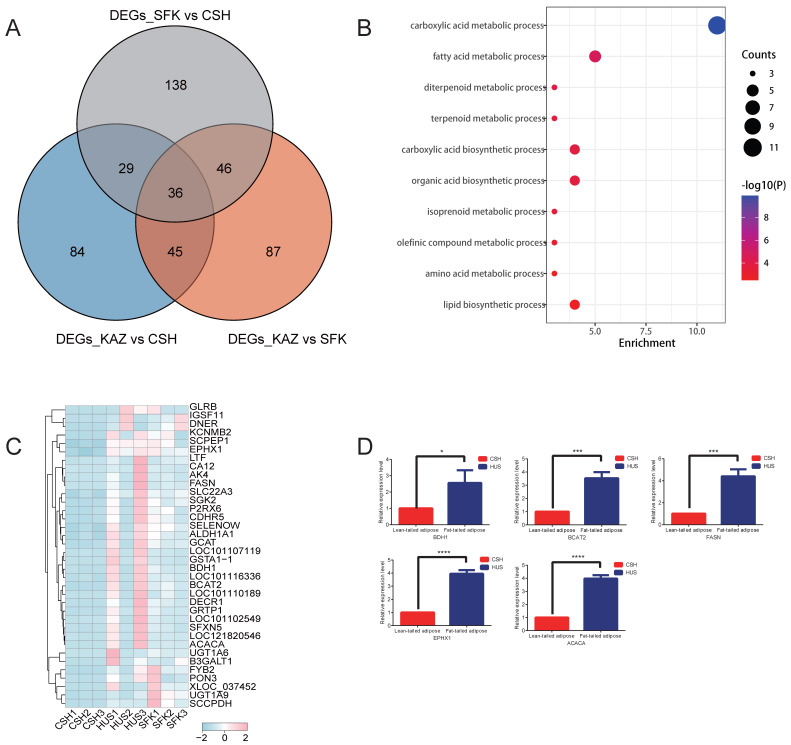
Identification of differentially expressed genes. (**A**) 36 overlap genes between KAZ and CSH, KAZ and SFK, and SFK and CSH groups. (**B**) 10 GO terms associated with lipid formation identified based on the 36 candidate genes. (**C**) Heatmap of 36 genes that co-located in both samples. (**D**) Gene expression levels of *BDH1*, *EPHX1*, *BCAT2*, *FASN*, and *ACACA* were determined by qRT-PCR and normalized to a housekeeping gene. Data are presented as mean ± SEM (or SD). Comparisons are between Lean-tailed, CSH (red bars) and Fat-tailed, KAZ (blue bars). Statistical significance is indicated by asterisks (*p < 0.05, *** *p* < 0.001, **** *p* < 0.0001), as determined by an unpaired *t*-test.

To further narrow down the key functional genes, 36 overlapping genes were identified across the three comparison groups (KAZ vs. CSH, KAZ vs. SFK, and SFK vs. CSH) (Figure 3A). Among these genes, *BDH1*, *EPHX1*, *BCAT2*, *FASN*, and *ACACA* are notably involved in fatty acid biosynthesis and metabolic processes, implying their potential role as core regulators of tail fat deposition. Furthermore, 10 Gene Ontology (GO) terms were annotated based on these 36 candidate genes (Table 1). Several critical pathways, including fatty acid metabolic process, lipid biosynthetic process, and carboxylic acid metabolic process, are confirmed to be closely associated with tail fat deposition in sheep (Figure 3B; Table 1). In addition, the differential expression patterns of these genes were also observed across the different comparison groups (Figure 3C). To validate the transcriptomic findings, qRT-PCR was performed on tail adipose tissue samples derived from the same experimental individuals. The qRT-PCR results revealed that all five candidate genes were significantly up-regulated in fat-tailed KAZ sheep compared with thin-tailed CSH sheep (Figure 3D), which was consistent with the RNA-sequencing (RNA-seq) data. These results further corroborate the involvement of these five genes in fatty acid biosynthesis and metabolic pathways, indicating that they may act as key regulators of tail fat deposition in sheep.

### 3.3. Analysis of lncRNA-Regulated Target Genes

**Figure 4:**
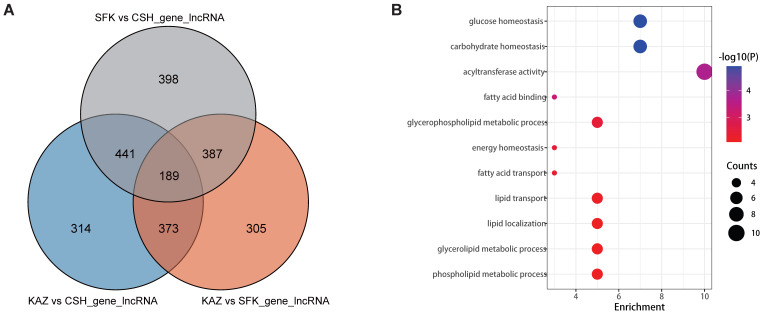
lncRNA regulated target genes. (**A**) 189 overlap target genes between KAZ and CSH, KAZ and SFK, and SFK and CSH groups. (**B**) 11 GO terms associated with lipid formation identified based on the candidate genes.

Elucidating the functions of non-coding RNAs remains a common challenge in RNA sequencing (RNA-seq) data analysis. Over the past few decades, long non-coding RNAs (lncRNAs) have been regarded as transcripts with minimal biological significance. Nevertheless, accumulating evidence indicates that lncRNAs are widely distributed in eukaryotes and play crucial roles in regulating gene expression, particularly in mammals. In the comparison between the KAZ and CSH groups, a total of 1317 lncRNA-regulated target genes were identified (Table S6). Similarly, 1254 lncRNA-regulated target genes were detected in the KAZ vs. SFK group comparison (Table S7). In the comparison between the SFK and CSH groups, 1415 lncRNA-regulated target genes were identified (Table S8). 189 overlap genes were identified among the three groups (Figure 4A). These genes play crucial roles in the formation and regulation of sheep tail fat. For instance, genes associated with the fatty acid transport system (*FABP4*, *FABP9*, and *FABP12*); those involved in lipid droplet formation and maintenance (*CLN3*, *AGPAT2*, and *OSBPL5*); and core genes regulating the balance of fat metabolism (*IGFBP5*, *SUCNR1*, *ADRB1*, and *VGF*). 11 GO terms associated with fat synthesis were identified, for instance, Fatty acid transport (including *FABP4*, *FABP9*, and *FABP12*), Lipid transport (including *CLN3*, *FABP4*, *OSBPL5*, *FABP9*, and *FABP12*), and Lipid localization (including *CLN3*, *FABP4*, *OSBPL5*, *FABP9*, and *FABP12*) (Figure 4B and Table 2).

### 3.4. Analysis of miRNA-Regulated Target Genes

**Figure 5:**
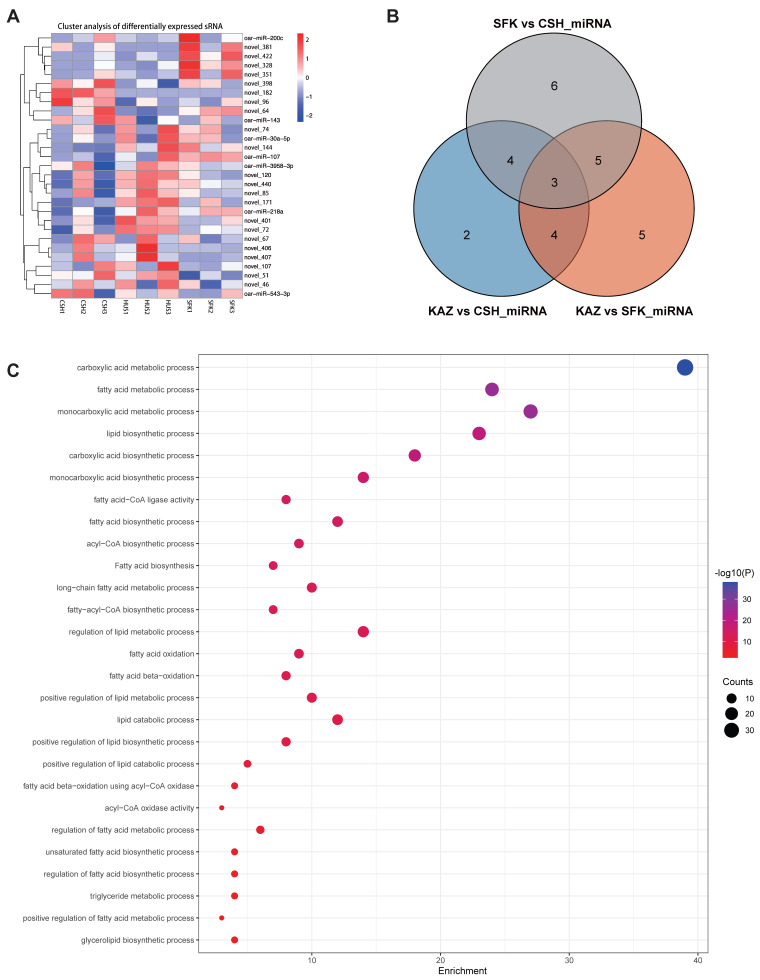
miRNA regulated target genes. (**A**) Heatmap of differential expressed miRNA, and red indicates high expression and blue indicates low expression. (**B**) 3 overlap miRNA between KAZ and CSH, KAZ and SFK, and SFK and CSH groups. (**C**) 27 GO terms associated with lipid formation identified based on 3 overlap miRNA regulated target genes.

Small RNAs (sRNAs) specifically recognize and bind to the RNA-induced silencing complex (RISC), thereby inhibiting target gene expression. sRNAs play pivotal roles in regulating nearly all cellular processes, including individual development, cell proliferation and differentiation, antiviral defense, and tumorigenesis. To further explore the potential functional roles of miRNAs in tail fat deposition, we analyzed a set of novel miRNAs across three pairwise comparisons (KAZ vs. CSH, KAZ vs. SFK, and SFK vs. CSH) (Figure 5A) Specifically, 13 miRNAs were identified in the KAZ vs. CSH comparison (Table 3), 17 miRNAs in the KAZ vs. SFK comparison (Table 4), and 18 miRNAs in the SFK vs. CSH comparison (Table 5). Three miRNAs (novel_120, novel_171, and novel_440) were detected in all three comparisons (Figure 5B and Table S9). Further analysis of the target genes of these three miRNAs led to the identification of 89 genes associated with fat synthesis and metabolism (Table S9), including genes involved in fat metabolism regulation (*LEPR*, *STAT3*, *ADRB1/2/3*, and *PPARγ*); those related to fatty acid synthesis (*ACACA* and *FASN*); and genes associated with fatty acid transport (the *ACSL* family and *ABCG1*) (Table S9). Based on the 89 candidate genes, 27 GO terms associated with fat synthesis were identified, for instance, lipid biosynthetic process, fatty acid metabolic process, lipid biosynthetic process, fatty acid-CoA ligase activity, and fatty acid oxidation (Figure 5C and Table S10).

## 4. Discussion

Lipid metabolism is governed by highly complex molecular mechanisms, and regulating fat deposition to optimize meat production traits holds great significance for sheep genetic improvement. To explore the genetic characteristics of adipose tissues and clarify breed-specific differences in the genetic regulatory networks underlying fat deposition, we systematically characterized the transcriptomic profiles of Kazakh sheep, Suffolk sheep, and their F2 hybrid generation (Figure 1). Several key differentially expressed genes (DEGs), long non-coding RNAs (lncRNAs), and novel miRNAs were identified.

### 4.1. Differentially Expressed Genes Related to Sheep Tail Fat Deposition

Firstly, our comprehensive transcriptomic profiling of tail adipose tissue across three sheep populations—KAZ, SFK, and CSH—uncovered pronounced differential gene expression patterns that underlie phenotypic variation in tail fat deposition (Figure 2A–C). A refined analysis of overlapping up-regulated genes between the two breeds (KAZ and SFK) relative to the thin-tailed CSH identified 36 shared genes (Figure 3A). Five genes, namely *ACACA*, *EPHX1*, *BCAT2*, *FASN*, and *BDH1*, were identified as key regulators of tail fat deposition in sheep, which is consistent with the findings of previous studies. *ACACA* was characterized as a differentially expressed gene (DEG) in the transcriptomic comparison of tail fat between Altay sheep and Small Tail Han sheep, where it participates in triglyceride biosynthesis [11]. Furthermore, the expression of *ACACA* is significantly upregulated in the tail fat of sheep across different growth stages, indicating that it serves as a key rate-limiting enzyme in de novo lipogenesis [44]. *EPHX1* has been repeatedly identified as a DEG in multiple transcriptomic studies focusing on sheep tail fat [10]. It is involved in the regulation of oxidative stress within the lipid metabolism network, and its elevated expression in the tail fat of fat-tailed sheep contributes to enhanced fat accumulation [11]. *BCAT2* was listed as a DEG in a meta-analysis of transcriptomic datasets associated with lipid metabolism in sheep tail fat. Notably, a novel splice variant of *BCAT2* is specifically expressed in the tail fat of fat-tailed sheep, which mediates branched-chain amino acid metabolism and thereby modulates fat deposition [45]. The mRNA and protein expression levels of *FASN* in the tail fat of fat-tailed sheep are significantly higher than those in thin-tailed sheep [46]. In adipocytes isolated from the tail fat of Altay sheep, *FASN* acts as a core gene governing fatty acid synthesis, and its expression is positively correlated with tail fat deposition [47]. Currently, direct research on the role of *BDH1* in sheep tail fat remains extremely limited. *BDH1* is a mitochondrial inner membrane-localized enzyme that catalyzes a key reversible reaction in ketone body metabolism: the interconversion between acetoacetate and β-hydroxybutyrate [48]. Its biological function is tightly linked to lipid metabolism, and its regulatory role has been verified in ruminant species. A study conducted on dairy goats demonstrated that *BDH1* exerts a negative regulatory effect on lipid metabolism in mammary epithelial cells; its overexpression leads to a significant reduction in intracellular triglyceride content and lipid droplet accumulation [49]. Another study revealed that exogenous supplementation of β-hydroxybutyrate during rumen development in lambs markedly upregulates the expression of lipolysis-related genes, with *BDH1* being included in this gene set [50]. Collectively, these findings suggest that *BDH1* not only participates in ketone body utilization but also directly or indirectly modulates the balance between lipogenesis and lipolysis.

In addition to the aforementioned well-characterized genes, several novel candidate genes potentially involved in sheep tail fat formation were identified in the present study. For instance, *SGK2* regulates the insulin signaling pathway, thereby affecting glucose and lipid metabolism, and participates in the modulation of adipocyte differentiation and lipogenesis [51]. *UGT1A6* catalyzes the metabolism of a variety of lipophilic substances and is involved in the metabolism of fatty acids and steroid hormones in sheep [52]. *DECR1* is an essential enzyme for fatty acid β-oxidation; it mediates the degradation of polyunsaturated fatty acids, regulates energy metabolism in adipose tissue, and affects the balance between fat storage and mobilization [51]. *SFXN5* modulates mitochondrial function and iron metabolism, which are closely associated with cellular energy production. By regulating mitochondrial energy metabolism in adipocytes, *SFXN5* exerts an impact on the efficiency of fat synthesis [38].

### 4.2. LncRNA Regulation of Target Genes of Sheep Tail Fat Deposition

Secondly, we analyzed the target genes regulated by long non-coding RNAs (lncRNAs) and distinguished between previously well-documented lncRNA-regulated target genes and newly identified ones, while clarifying their roles in key biological pathways.

Among the known genes identified in our lncRNA target analysis, the *FABP4* gene—regulated by two lncRNAs (TCONS_00111676 and TCONS_00111678)—plays a central role in the intracellular transport, distribution, and metabolic regulation of fatty acids [53]. As one of the most well-studied members of the *FABP* family, *FABP4* is highly expressed primarily in adipocytes and macrophages [54], and numerous studies have confirmed that its expression level is closely associated with adipocyte differentiation and lipid accumulation [55]. For instance, in bovine mammary epithelial cells, *FABP4* has been validated to regulate milk fat metabolism, with its upregulation promoting triglyceride formation and lipid droplet assembly [56]. Similarly, in caprine mammary epithelial cells, *FABP4* expression is modulated by *PPARD* (a nuclear receptor maintaining lipid homeostasis) [57]. Extrapolating from these findings, *FABP4* is inferred to also play a critical role in sheep tail adipocytes by facilitating efficient fatty acid transport into cells for triglyceride synthesis. Additionally, other known *FABP* family members, such as *FABP9* and *FABP12*, may be involved in this process, collectively forming an efficient fatty acid transport network that provides the material basis for rapid tail fat deposition [58].

Furthermore, after intracellular fatty acids are converted into neutral lipids (primarily triglycerides), they are stored in organelles called lipid droplets (LDs) [59]. LDs are dynamic organelles whose formation, fusion, growth, and breakdown are tightly regulated at the molecular level [60]; LD fusion, a key mechanism for volume expansion, involves complex energy pathways and lipid component regulation [61]. In our study, we identified three novel lncRNA-regulated genes—*AGPAT2*, *OSBPL5*, and *CLN3*—that play important roles in LD formation and maintenance. *AGPAT2* (1-acylglycerol-3-phosphate O-acyltransferase 2) is a key enzyme in the triglyceride synthesis pathway, catalyzing the conversion of lysophosphatidic acid to phosphatidic acid and thereby providing substrates for triglyceride backbone formation [62]. *OSBPL5* (oxysterol-binding protein-related protein 5) may participate in inter-organelle lipid transport (e.g., lipid transfer between the endoplasmic reticulum and LDs), thereby influencing LD growth and composition [63]. Although *CLN3* is primarily known to be associated with neuronal ceroid lipofuscinosis, its encoded protein localizes to the lysosomal and endosomal systems—organelles involved in LD breakdown via “lipophagy” [64]. *TKAZ*, our novel finding suggests that *CLN3* may indirectly regulate net fat accumulation by modulating LD turnover rate. Collectively, these newly identified genes, together with the known *FABP* family members, ensure efficient fat synthesis and secure LD storage to support sheep tail fat deposition.

### 4.3. miRNA-Regulated Target Genes of Sheep Tail Fat Deposition

Thirdly, our study innovatively analyzed miRNA-regulated target genes and identified three overlapping miRNAs (novel_120, novel_171, and novel_440) across the three comparisons, which represents a key novel finding of this research (Figure 5B and Table S9). A critical breakthrough of our work lies in the further identification that novel_171, one of these overlapping miRNAs, directly targets a set of adipogenesis-related genes, including *PPARγ*, *ACACA*, *FASN*, *LPL*, and *SIRT1* (Table S9).

Notably, these novel_171-targeted genes are well-recognized as core regulators of adipogenesis and lipid metabolism: Peroxisome proliferator-activated receptor γ (*PPARγ*) is a master regulator of adipocyte differentiation; acetyl-CoA carboxylase (*ACACA*) and fatty acid synthase (*FASN*) are rate-limiting enzymes in de novo fatty acid synthesis; lipoprotein lipase (*LPL*) mediates circulating triglyceride hydrolysis to supply fatty acids for adipocytes; and sirtuin 1 (*SIRT1*), an NAD+-dependent deacetylase, modulates energy metabolism and inhibits adipogenesis. While previous studies have reported the roles of these genes in adipogenesis (e.g., *PPARγ* regulated by miR-433-3p/485-3p in ovine preadipocytes [65], *ACACA* inhibited by miR-151b [48], *LPL* associated with sheep tail fat deposition [66,67], and *SIRT1* targeted by oar-miR-432 [68]), our study is the first to demonstrate that all these key genes are integrated into a single regulatory network controlled by the novel miRNA (novel_171), providing a new regulatory axis underlying adipogenesis. Another novel insight from our study is that these novel_171-targeted genes are significantly enriched in several critical biological pathways closely related to fat deposition, including carboxylic acid metabolism, fatty acid synthesis and degradation, and triglyceride synthesis (Table S10 and Figure 5C). These pathways constitute the core of lipid biosynthesis and storage: de novo fatty acid synthesis converts small-molecule carbon sources (primarily acetyl-CoA) into long-chain fatty acids [69], while triglyceride synthesis (the final form of fat storage) esterifies fatty acids to glycerol backbones to form neutral triglycerides (TAGs) stored in adipocyte lipid droplets [70]. Our findings link the novel_171-target gene network to these core metabolic pathways, revealing a new molecular mechanism that coordinates sheep tail fat deposition—a complex biological process driven by the balance between fatty acid/glyceride biosynthesis (anabolic pathways) and catabolic pathways such as fatty acid β-oxidation.

### 4.4. Limitations of This Study

Firstly, we acknowledge a key limitation of this study: the small sample size (*n* = 3 per group) for transcriptomic profiling of tail fat tissues. This substantially constrains statistical power, as smaller sample sizes reduce the ability to distinguish true biological variations from random noise, thereby increasing the risk of false positives or false negatives among the identified DEGs. Additionally, it raises the potential for overinterpretation of the observed differential expression patterns—including the core candidate genes and the lncRNA-mRNA-miRNA regulatory network. Future studies should expand the sample size and incorporate additional biological replicates to validate the robustness of the identified pathways, which will enhance the reliability of findings and their translational value for sheep genetic improvement targeting tail fat traits.

A potential another limitation of this study is the confounding effect of sex on adipose tissue gene expression, as our experimental cohort included KAZ ewes, SFK rams, and CSH. Sex dimorphism is a well-documented modulator of transcriptomic profiles in adipose tissue, driven by the differential actions of sex hormones: estrogen promotes adipocyte differentiation and lipid accumulation, while testosterone tends to inhibit adipogenesis and enhance lipolysis. Future studies with sex-matched experimental groups are warranted to further dissect the breed-specific regulatory mechanisms of adipose tissue development independent of sex effects.

In addition, FPKM method is affected by both gene length and sequencing depth. For genes with extreme lengths, their FPKM values may overestimate or underestimate the actual expression level, and direct cross-sample comparison of FPKM values may introduce false positives. The alignment sensitivity of TopHat is lower than that of current tools such as HISAT2 and STAR, especially for transcripts with complex alternative splicing patterns. Cuffdiff also has limited statistical power in identifying low-abundance differentially expressed genes compared with DESeq2/edgeR. Furthermore, this study conducted bioinformatics analysis to predict the lncRNA-mRNA and miRNA-mRNA regulatory relationships related to sheep tail fat deposition, and screened out core target genes such as *FABP4*, *PPARγ*, and *ACACA* involved in lipid metabolism.

Furthmore, it should be noted that all the current regulatory relationships are merely prediction results and have not yet been confirmed through functional verification methods such as knockdown/overexpression or luciferase reporter gene experiments. Experimental verification is crucial for confirming the authenticity of molecular interactions. The absence of this step may lead to false positives in the prediction results and affect the reliability of the regulatory network analysis. Therefore, subsequent studies should prioritize functional verification of the core regulatory factors to clarify their actual mechanism of action in tail fat deposition.

## 5. Conclusions

This study investigated the molecular regulatory mechanisms underlying tail fat deposition in fat-tailed KAZ sheep, thin-tailed SFK sheep, and CSH sheep. Differential expression analysis identified significant differentially expressed genes (DEGs) between KAZ and CSH, KAZ and SFK, and SFK and CSH groups. Core genes regulating tail fat deposition, including *BDH1*, *EPHX1*, *BCAT2*, *FASN*, and *ACACA*, were found to be involved in fatty acid synthesis and metabolism pathways, with qRT-PCR confirming their upregulation in fat-tailed sheep. Additionally, 189 lncRNAs targeting key genes (e.g., *FABP* family, *AGPAT2*) and 3 common differentially expressed miRNAs (novel_120, novel_171, novel_440) were identified, with their target genes enriched in lipid synthesis-related GO terms and pathways. The study confirmed that the LncRNA-mRNA-miRNA regulatory axis is a crucial pathway in tail fat formation, providing important molecular targets and theoretical basis for genetic improvement of tail fat traits in sheep.

## Figures and Tables

**Table 1:** Top 10 GO terms based on candiated 36 genes.

ID	−log10(*p*)	Counts	Genes
carboxylic acid metabolic process	9.9	11	*ACACA*|*ALDH1A1*|*BCAT2*|*DECR1*|*EPHX1*|*FASN*|*GSTA1*|*PON3*|*GCAT*|*UGT1A9*|*SCPEP1*
fatty acid metabolic process	4.7	5	*ACACA*|*DECR1*|*EPHX1*|*FASN*|*GSTA1*
diterpenoid metabolic process	4	3	*ALDH1A1*|*UGT1A9*|*SCPEP1*
terpenoid metabolic process	3.8	3	*ALDH1A1*|*UGT1A9*|*SCPEP1*
carboxylic acid biosynthetic process	3.8	4	*ACACA*|*ALDH1A1*|*BCAT2*|*FASN*
isoprenoid metabolic process	3.6	3	*ALDH1A1*|*UGT1A9*|*SCPEP1*
olefinic compound metabolic process	3.3	3	*ALDH1A1*|*EPHX1*|*GSTA1*
amino acid metabolic process	2.6	3	*ALDH1A1*|*BCAT2*|*GCAT*
lipid biosynthetic process	2.5	4	*ACACA*|*FASN*|*B3GALT1*|*SCCPDH*
organic acid biosynthetic process	3.8	4	*ACACA*|*ALDH1A1*|*BCAT2*|*FASN*

**Table 2:** Top 11 GO terms based on candiated 189 genes.

ID	−log10(*p*)	Counts	Genes
glucose homeostasis	4.9	7	*IGFBP5*|*PYGL*|*VGF*|*RACK1*|*FIS1*|*CCDC186*|*SUCNR1*
carbohydrate homeostasis	4.9	7	*IGFBP5*|*PYGL*|*VGF*|*RACK1*|*FIS1*|*CCDC186*|*SUCNR1*
acyltransferase activity	3.7	10	*MDM2*|*TRIM27*|*AGPAT2*|*FBXO22*|*TRIM7*|*TRIM52*|*TRIM41*|*UBE2Q2*|*TRIM9*|*NAT16*
fatty acid binding	3.3	3	*FABP4*|*FABP9*|*FABP12*
glycerophospholipid metabolic process	2.5	5	*CLN3*|*INPP4B*|*AGPAT2*|*OSBPL5*|*ABHD12B*
energy homeostasis	2.5	3	*ADRB1*|*VGF*|*SUCNR1*
fatty acid transport	2.4	3	*FABP4*|*FABP9*|*FABP12*
lipid transport	2.2	5	*CLN3*|*FABP4*|*OSBPL5*|*FABP9*|*FABP12*
lipid localization	2.1	5	*CLN3*|*FABP4*|*OSBPL5*|*FABP9*|*FABP12*
glycerolipid metabolic process	2.1	5	*CLN3*|*INPP4B*|*AGPAT2*|*OSBPL5*|*ABHD12B*
phospholipid metabolic process	2.1	5	*CLN3*|*INPP4B*|*AGPAT2*|*OSBPL5*|*ABHD12B*

**Table 3:** miRNA identified in KAZ vs. CSH.

sRNA	KAZ_Readcount	CSH_Readcount	log_2_ Fold Change	*p*_adj_
novel_120	81.12688766	18.25589902	2.113645506	0.019040978
novel_144	32.50577511	2.954650549	3.515775872	0.000415888
novel_171	16.14543989	3.222043669	2.361046738	0.024388178
novel_182	0	6.63064167	−5.220338598	0.008141076
novel_401	35.55721207	12.73142355	1.458682151	0.047982783
novel_440	401.4417819	153.3723361	1.382264301	0.037029294
novel_64	340.9949191	1378.204568	−2.014920104	0.001494234
novel_72	942.3554226	350.5386447	1.42563343	0.003351532
novel_74	738.9185669	269.494113	1.453967425	0.020565608
novel_96	117.3840151	339.4617277	−1.529354637	0.006081166
oar-miR-200c	12.55231807	50.1763364	−1.998804459	0.034227956
oar-miR-218a	3513.564379	939.5928252	1.902250511	0.0009257
oar-miR-30a-5p	286,156.3684	120,173.3036	1.251682203	0.049104905

**Table 4:** miRNA identified in KAZ vs. SFK.

sRNA	KAZ_Readcount	SFK_Readcount	log_2_ Fold Change	*p*_adj_
novel_107	187.4774452	47.23145136	1.988532171	0.00746145
novel_120	86.19938695	14.29460596	2.586105397	3.87 × 10^−5^
novel_171	17.29327335	5.636296059	1.635596866	0.046322273
novel_328	2.780490469	13.62539798	−2.308408787	0.026557759
novel_351	30.74939092	81.311312	−1.41142447	0.026922407
novel_381	0	31.76706191	−7.39025532	1.39 × 10^−5^
novel_401	37.98952298	14.1001242	1.422345668	0.023677844
novel_406	26.0639549	0.644637531	5.350917278	0.001776704
novel_407	12.01953184	0.318223692	5.13545176	0.004352336
novel_422	1.796100521	22.29467497	−3.549496518	0.001878239
novel_440	421.6404767	178.3429071	1.241322205	0.006960603
novel_51	1537.573428	871.7198229	0.819062658	0.016961335
novel_64	365.9416972	815.3732463	−1.156710525	0.003061472
novel_72	1006.41901	362.6546951	1.472635288	0.000182182
novel_85	568.2580957	190.1219305	1.579790419	0.001040954
oar-miR-200c	13.55313675	131.7782421	−3.287808686	0.004145702
oar-miR-3958-3p	1634.372414	1030.286099	0.665885251	0.034072262

**Table 5:** miRNA identified in SFK vs. CSH.

sRNA	SFK_Readcount	CSH_Readcount	log_2_ Fold Change	*p*_adj_
novel_107	45.11340409	162.445464	−1.844592802	0.012358134
novel_120	80.12688746	16.25549502	2.513666506	0.017045779
novel_144	20.20639307	3.122746156	2.761593211	0.006108951
novel_171	34.14443949	13.42404347	2.441046744	0.024484824
novel_182	0	6.966549737	−5.362681242	0.004130829
novel_398	23,527.0423	46,402.41249	−0.979861892	0.030305626
novel_406	0.61215494	9.059214455	−3.922624283	0.027163669
novel_407	0.30610738	6.357457678	−4.306032884	0.024302405
novel_422	21.28621329	4.171512318	2.421165892	0.021198952
novel_440	301.4417822	53.34556778	1.382264301	0.033942478
novel_46	977.019282	1825.176219	−0.901242653	0.023611069
novel_51	831.1719688	2141.954377	−1.365475571	0.005094795
novel_67	512.966282	945.8180573	−0.882024792	0.034356574
novel_96	119.5784418	357.6641415	−1.577791523	0.001868065
oar-miR-107	658.8736287	341.3256278	0.9496936	0.014890752
oar-miR-143	3,663,424.793	7,103,784.98	−0.955396879	0.036211056
oar-miR-218a	2290.135514	975.9617483	1.229477253	0.009048071
oar-miR-543-3p	28.74588129	70.48302285	−1.294548206	0.044386631

## Data Availability

RNA-seq data have been deposited in the GEO database (GEO: GSE290403) and are publicly available as of publication. This study does not report original algorithms. Any additional information required to reanalyze the data reported in this paper is available from the lead contact upon request.

## Supplementary Materials

The following supporting information can be downloaded at: https://www.mdpi.com/article/10.3390/genes17020162/s1. Table S1: Primer sequences and amplification characteristics of candidate genes in sheep. Table S2: Statistics of total reads and mapped reads. Table S3: Analysis of DE genes of KAZ vs. CSH. Table S4: analysis of DE genes of KAZ vs. SFK. Table S5: analysis of DE genes of SFK vs. CSH. Table S6: lncRNA idenfited in KAZ_vs_CSH. Table S7: lncRNA idenfited in KAZ_vs_SFK. Table S8: lncRNA idenfited in SFK_vs_CSH. Table S9: Genes identified based on overlap 3 miRNA. Table S10: GO terms identifed by 89 candiated genes.

## Abbreviations

DEGs	Differentially Expressed Genes
qRT-PCR	Quantitative Real-Time Polymerase Chain Reaction
KEGG	Kyoto Encyclopedia of Genes and Genomes
FPKM	Fragments Per Kilobase of transcript per Million mapped reads
lncRNAs	Long Non-Coding RNAs
miRNAs	MicroRNAs
ncRNAs	Non-Coding RNAs
GO	Gene Ontology
BAM	Binary Alignment/Map
SAM	Sequence Alignment/Map
GTF	Gene Transfer Format
RISC	RNA-Induced Silencing Complex
SBS	Sequencing by Synthesis
WAT	White Adipose Tissue
BCAA	Branched-Chain Amino Acid
BCKA	Branched-Chain α-Keto Acids
EETs	Epoxyeicosatrienoic Acids
LDs	Lipid Droplets
TAGs	Triacylglycerols
PPARγ	Peroxisome Proliferator-Activated Receptor γ
LPL	Lipoprotein Lipase
BMP2	Bone Morphogenetic Protein 2
ASE	Allele-Specific Expression
SIRT1	Sirtuin 1
SEM	Standard Error of the Mean
SD	Standard Deviation
GEO	Gene Expression Omnibus
CPC2	Coding Potential Calculator 2
CNCI	Coding-Non-Coding Index
HGNC	HUGO Gene Nomenclature Committee
RNase	Ribonuclease
ANOVA	Analysis of Variance
